# Renal Manifestations of Chronic Hepatitis C: A Review

**DOI:** 10.3390/jcm13185536

**Published:** 2024-09-18

**Authors:** Aalam Sohal, Carol Singh, Akshita Bhalla, Harsimran Kalsi, Marina Roytman

**Affiliations:** 1 Division of Gastroenterology and Hepatology, Creighton University School of Medicine, Phoenix, AZ 2500, USA; 2 Department of Internal Medicine, Dayanand Medical College and Hospital, Ludhiana 141001, Punjab, India; singhcarol04@gmail.com; 3 Department of Internal Medicine, Punjab Institute of Medical Sciences, Jalandhar 144006, Punjab, India; akshitabhalla11@gmail.com; 4 Department of Internal Medicine, University of Central Florida College of Medicine, Orlando, FL 32827, USA; simrankalsi407@gmail.com; 5 Division of Gastroenterology and Hepatology, University of California San Francisco, Fresno, CA 93701, USA; marina.roytman@ucsf.edu

**Keywords:** hepatitis C virus, renal manifestations, glomerulonephritis, vasculitis, chronic kidney disease, dialysis, kidney transplantation, nephrotoxicity

## Abstract

Hepatitis C virus (HCV) has emerged as a major global health concern and, if left untreated, can lead to significant liver damage, including cirrhosis, decompensated liver disease, and hepatocellular carcinoma (HCC). Approximately 40% of patients with HCV infection experience extrahepatic manifestations, including renal involvement. HCV-related renal disease is of significant importance among patients with chronic kidney disease (CKD), leading to higher morbidity and mortality. The renal damage due to HCV infection primarily results from cryoglobulinemia and glomerulonephritis, with conditions such as membranoproliferative glomerulonephritis (MPGN) and membranous nephropathy (MN) being most prevalent. Despite advancements in treatment, including the use of directly acting antiviral agents (DAAs), renal complications remain a significant burden in untreated patients. HCV-positive patients on hemodialysis (HD) or those who have undergone kidney transplantation face increased mortality rates compared to their HCV-negative counterparts. Managing HCV infection before kidney transplantation is crucial to mitigate the risk of HCV-related renal complications. Conversely, kidney transplantation from HCV-infected donors is well established, as post-transplant treatment for HCV is safe and effective, potentially reducing mortality and morbidity for patients on transplant waiting lists. This review aims to provide a comprehensive analysis of the renal manifestations of HCV, emphasizing the importance of early diagnosis and treatment to improve patient outcomes.

## 1. Introduction

Hepatitis C virus (HCV) is a significant global health issue that affects more than 50 million people worldwide and leads to 1 million new infections annually, as reported by the WHO [1]. HCV, first isolated in 1989, is an enveloped, positive-strand RNA virus that belongs to the Flaviviridae family [2,3,4]. It is known to cause progressive liver damage ranging from acute to chronic hepatitis, cirrhosis, decompensated liver disease, and hepatocellular carcinoma (HCC) [5].

HCV is transmitted through various modes, including unscreened blood and blood products, injection drug use, non-sterile injections, unsafe medical practices in healthcare environments, via sexual contact, from mother to child during childbirth, and through certain cosmetic procedures [6]. The modes of HCV transmission have changed significantly over time [6]. Historically, the virus was primarily spread through blood transfusions and iatrogenic transmission [6]. Today, in high-income countries, the risk of HCV transmission through blood transfusions has been effectively controlled, and iatrogenic exposure has been largely reduced [6]. In contrast, blood transfusions and non-sterile injection practices are currently the major contributors to the spread of HCV in low-income countries, highlighting global disparities [6]. Currently, injection drug use is the main driver of infection, particularly in high-income countries and regions where blood products are routinely screened for HCV [1,6,7]. Emerging high-risk groups include men who have sex with men (MSM), individuals who undergo tattoo procedures, and newborns of HCV-infected pregnant women [6]. Cosmetic procedures like tattooing and body piercing pose a risk for HCV transmission when instruments contaminated with infectious blood are not properly sterilized, leading to the potential spread of the virus to others [6].

Approximately 40% of individuals with chronic HCV infection experience at least one extrahepatic manifestation during their illness [8]. These extrahepatic manifestations can affect nearly every organ system and include metabolic syndromes (such as diabetes mellitus), cardiovascular complications (cerebrovascular accident, acute myocardial infarction), autoimmune diseases (such as Sjogren’s syndrome, thyroiditis, and arthritis), immune-mediated disorders (such as mixed cryoglobulinemia, polyarteritis nodosa (PAN), monoclonal gammopathies, immune thrombocytopenia), malignancies (including B-cell lymphoma), dermatologic conditions (such as lichen planus, porphyria cutanea tarda, pruritus), and organ-specific (renal, pulmonary, ocular) and neuropsychiatric conditions (depression, cognitive impairment) [9,10,11,12,13].

HCV infection is known to cause certain types of renal disease and exacerbate others [14,15]. Kidney damage among patients with HCV occurs due to cryoglobulinemia and glomerulonephritis [16,17]. The prevalence of cryoglobulinemia has been reported to be 2–4 times higher in HCV than in hepatitis B virus (HBV) [18,19]. Other renal manifestations include membranoproliferative glomerulonephritis (MPGN), membranous nephropathy (MN), and, rarely, antineutrophil cytoplasmic antibody (ANCA)-associated glomerulonephritis [20,21].

The presence of HCV in patients with chronic kidney disease (CKD) has been linked to a higher risk of morbidity and mortality [22]. Although the introduction of directly acting antiviral agents (DAAs) has significantly reduced the morbidity and mortality rates, renal complications in untreated patients pose a significant burden [23]. Kidney transplant recipients or patients on hemodialysis (HD) who are HCV-positive have higher mortality rates compared to HCV-negative patients [24,25]. HCV infection should be treated before kidney transplantation to mitigate the risk of HCV-related renal complications in the transplanted kidney [24]. However, kidney transplant candidates on the waiting list are advised to consider accepting donors who are HCV antibody-positive and NAT-positive, as post-transplant treatment for HCV infection is both safe and effective [26]. Declining organs from HCV-infected donors could lead to increased mortality and morbidity rates for patients on the kidney transplant waiting list [26].

This paper aims to offer a comprehensive review and detailed analysis of the renal manifestations of HCV infection to improve the understanding and management of its complications.

## 2. Methodology

The objective of conducting this literature review was to assess the various renal complications seen in patients with chronic hepatitis C patients. Various databases, including PubMed, Google Scholar, EMBASE, and MEDLINE, were used to locate sources for this literary analysis. Search terms used for this review consisted of Hepatitis C virus; Renal manifestations; Glomerulonephritis; Vasculitis; Chronic kidney disease; Dialysis; Kidney transplantation; and Nephrotoxicity. The studies were reviewed by two independent authors, and if there was any conflict regarding inclusion/exclusion of the study in the literature review, the opinion of the third author was taken into account.

### 2.1. HCV-Related Glomerulopathies and Vasculitis

#### 2.1.1. HCV-Related Glomerulopathies

Studies by Johnson et al. and Agnello et al. carried out in 1992–1993 were some of the first to document an association between HCV and glomerular disease [27,28]. Most of the cases reported by these studies were suggestive of mixed cryoglobulinemia and MPGN, but the etiopathogenesis behind these was not clearly understood [27]. Although the cause was previously unknown, HCV infection has now been identified as responsible for 80–90% of mixed cryoglobulinemia cases [17,28,29,30].

Recent large-scale studies have shown that the incidence of HCV-associated glomerulonephritis is relatively low. Moorman et al. reported that only 0.3% of a large cohort of patients with HCV viremia exhibited nephrotic syndrome. In the same group, cryoglobulinemia was observed in 0.9% of patients [31]. These findings align with data from the Nationwide Inpatient Sample, which reported the occurrence of nephrotic syndrome or MPGN in this cohort to be 0.38% [32].

The most frequent HCV-associated glomerulopathy responsible for renal impairment is Type 1 MPGN, which occurs as a result of the most common HCV-related small vessel vasculitis namely mixed cryoglobulinemia [33,34]. Although MPGN is most frequently linked with HCV infection, various other histological types of renal diseases are reported in association with HCV infection, including MN, focal segmental glomerulosclerosis (FSGS), mesangial proliferative glomerulonephritis (mesPGN), fibrillary glomerulonephritis, immunotactoid glomerulopathy, IgA nephropathy (IgAN), and renal thrombotic microangiopathy [30,35,36,37,38]. Other glomerular diseases, such as diabetic nephropathy, are also often reported among patients with HCV infection, which may be partially attributed to the higher incidence of diabetes in HCV-infected patients compared to the general population [37,39,40,41,42].

##### Pathogenesis of HCV-Related Glomerulopathies

i **Cryoglobulinemic Glomerulonephritis**

HCV may affect the kidneys through three mechanisms: formation of immune complexes, cryoglobulins, and the virus’s direct cytopathic effect [43].

HCV is primarily associated with mixed cryoglobulinemia and cryoglobulinemic vasculitis, which is a systemic vasculitis mainly affecting small–medium-sized blood vessels [44,45]. Cryoglobulins are subdivided into three types: type I (isolated monoclonal immunoglobulin), type II (IgG and an IgM rheumatoid factor (RF) of monoclonal origin/mixed cryoglobulinemia), and type III (IgG and a polyclonal IgM RF) [46]. The factors predisposing HCV-infected patients to develop mixed cryoglobulinemic vasculitis are not fully understood, but the host’s immune response genes may play a role [47]. Chronic HCV infection, which accounts for 80–90% of cases of mixed cryoglobulinemia, is the main cause of the formation of type II and type III mixed cryoglobulinemia [48].

Deposition of immune complexes in small blood vessels and subsequent endothelial injury leads to cryoglobulinemic vasculitis [49]. In HCV-associated mixed cryoglobulinemia, anti-HCV polyclonal IgG, and monoclonal IgM with RF and chronic B-cell clones are formed [50]. These cryoprecipitable immune complexes evade the erythrocyte transport system. The presence of IgM induces complement activation and consumption but fails to incorporate complement fragments, including complement C3b, the binding of which in turn favors the binding of the immune complexes to the erythrocyte complement receptor 1 (CR1) [51]. As a result, these immune complexes circulate in blood, not only because of evasion of the erythrocyte transport system but also because hepatic and splenic macrophages cannot process them [52]. Another study by Roccatello suggests that the impaired handling of cryoglobulins by the liver and, to a lesser extent, the spleen may contribute to the pathogenesis of essential mixed cryoglobulinemic glomerulonephritis [51]. The relative deficiency of cryoglobulin binding to erythrocytes may prolong their circulation plasma phase, increasing the chances of cryoglobulin deposition in different tissues [53]. Studies suggest that the circulating immune complexes localize to the glomerular capillaries. Subendothelial and mesangial deposition then leads to local cellular proliferation and leukocyte infiltration [27]. The attempts made by the phagocytes to remove deposited cryoglobulins potentially perpetuate glomerular damage [52]. HCV may also promote the development of lymphoma through persistent antigenic stimulation and its direct oncogenic potential [54]. Other autoimmune diseases may also occur, including the increased risk of non-Hodgkin lymphoma, which may contribute to renal damage and impact the overall prognosis [43,55].

ii **Non-Cryoglobulinemic Glomerulonephritis**

Non-cryoglobulinemic glomerulonephritis occurs without circulating cryoglobulin and may involve the deposition of immune complexes formed by HCV and IgG [13]. Glomerular diseases linked with non-cryoglobulinemic glomerulonephritis include non-cryoglobulinemic MPGN, mesPGN, MN, IgAN, FSGS, and fibrillary-immunotactoid glomerulopathy [30,33,43].

##### Characteristics of Various Glomerular Diseases

  **1.**  **Membranoproliferative Glomerulonephritis**

The primary glomerular lesion associated with HCV infection is cryoglobulinemic MPGN [37]. Type 1 MPGN is the most common presentation of HCV and occurs because of the precipitation of type 2 mixed cryoglobulins [27]. Non-cryoglobulinemic immune complexes may also form deposits, and anti-HCV IgG deposits and complement fragments may be found in the mesangium, leading to non-cryoglobulinemic MPGN [29,37,56].

The renal manifestation of MPGN due to cryoglobulinemia differs from those of idiopathic MPGN or due to SLE [57]. Light microscopy reveals monocytic infiltration resulting in endocapillary proliferation and infiltration, which occurs more in the case of cryoglobulinemia [58]. Peripheral interposition of monocytes results in double-contoured appearance of the basement membrane [59]. Electron microscopy shows subendothelial deposits that have tubular and crystalline patterns [60]. Immunofluorescence reveals prominent IgM and C3 staining on the capillary walls [61]. Additionally, intracapillary thrombi often show positive staining for IgM and either clonal κ or λ chains [61].

Renal biopsies in patients with non-cryoglobulinemic membranoproliferative glomerulonephritis show mesangial proliferation and sclerosis, increased cellularity, accentuation of glomerular lobular structure, tubular atrophy, infiltration of mononuclear cells, and a double contour of the basement membrane [62]. Additionally, immunofluorescence reveals the deposition of IgM, IgG, and complement (primarily C3) within the mesangium and capillary walls [62].

Cryoglobulinemic glomerulonephritis presents clinically with a combination of the Meltzer triad, which includes skin vasculitis, arthralgia, and myalgia, along with symptoms indicative of CKD [43,63,64]. CKD may manifest as asymptomatic hematuria and/or proteinuria as well as progressive renal failure [43]. Nephrotic syndrome is observed in about 20% of cases, while nephritic syndrome occurs in approximately 16%. Additionally, hypertension is present in around 70% of patients [43].

  **2.**  **Membranous Nephropathy**

MN may also occur in cases of HCV infection, but MN is more commonly associated with HBV infection [65,66]. Co-infection with HCV and HBV in patients with MN has also been documented [67]. A study by Stehman-Breen et al., conducted in 1995, reported four patients with membranous glomerulonephritis and HCV infection, with normal or minimally reduced complement levels and no evidence of rheumatoid factor or cryoglobulinemia [68].

The pathology of HCV-related MN is similar to that of idiopathic MN [37]. Serum complement levels are typically normal, and cryoglobulins and rheumatoid factors are absent in the serum [37,69]. In a study by Yamabe et al. in which 146 patients with various types of glomerulonephritis and renal diseases were monitored between 1990 and 1993, 8.3% of patients with MN were observed to have anti-HCV antibodies or detectable HCV RNA [70]. The mechanism of renal injury in HCV-related MN is believed to be due to subepithelial glomerular deposition of immune complexes containing HCV proteins [33]. Light microscopy typically reveals a diffuse thickening of the glomerular basement membrane without mesangial proliferation [71]. Diffuse and granular deposits of IgG, C3, and IgA are seen on immunofluorescence [71]. Electron microscopy reveals diffuse subepithelial immune deposits [71].

  **3.**  **Focal Segmental Glomerulosclerosis**

Unlike the more common cryoglobulin-induced glomerulopathy, FSGS is a rarer manifestation of renal disease in HCV-infected individuals [33,72]. An association between HCV infection and FSGS was observed in a study conducted by Altraif et al. in 1995 [73]. Similarly, Stehman-Breen et al. carried out a study in 1999 suggesting that HCV infection might contribute to the development of FSGS in susceptible individuals and that chronic HCV infection could be involved in the pathogenesis of heroin nephropathy [72]. Another study conducted in 2005 in Egypt by Sabry et al. with the aim to describe the histological pattern of HCV-associated nephropathy on histological examination reported MPGN and FSGS as the most common lesions observed, accounting for 39% and 30%, respectively, among 233 patients who presented with glomerulopathy [74]. Cases documenting the rare association of FSGS and HCV have been reported by Ezaki Y et al. (1999), Motta M et al. (2001), Sperati CJ et al. (2013), and Shah HH et al. (2013) [75,76,77,78].

FSGS is an antibody-independent glomerulonephritis [33]. The fundamental pathogenic mechanisms are not well understood, but it is hypothesized that HCV, similar to human immunodeficiency virus (HIV), directly damages podocytes, leading to segmental glomerulosclerosis [79]. Patients may present with symptoms of primary FSGS, complicating the diagnosis; however, laboratory and clinical features of HCV infection assist in distinguishing between the two types [80].

FSGS, when classified histologically, includes five different variants [81,82,83]. In cases induced by viruses, the collapsing type is the most frequently observed [84]. An emergence of cases of de novo collapsing FSGS (cFSGS) was observed during the use of interferon therapy for treatment of HCV infection. A combination of interferon alfa-2a and ribavirin has been used previously to stabilize HCV-associated cFSGS [75,76,77,78].

  **4.**  **Mesangial Proliferative Glomerulonephritis**

MesPGN has been occasionally seen in patients with chronic HCV infection [85]. On renal biopsy in HCV patients, roughly 80% of the patients exhibit diffuse MPGN, 10% reveal focal MPGN, and another 10% of patients exhibit mesPGN [86]. MesPGN is characterized by diffuse mild mesangial expansion and increased mesangial cellularity [85].

  **5.**  **IgA Nephropathy**

Some cases showing an association between IgAN and HCV infection have also been reported in literature [87,88,89,90,91]. Serum IgA levels rise in cirrhotic patients as a result of decreased hepatic clearance of IgA immune complexes due to hepatic insufficiency in HCV infection, which increases the likelihood of IgA deposition in the nephron glomeruli [92]. IgA deposition in the glomeruli is seen in most forms of cirrhosis [33,92,93]. Therefore, HCV may not be directly involved in the pathogenesis of the disease, and it is not the only factor linked to the development of IgAN in cirrhotic patients [33,92,94].

This secondary form of IgAN varies in severity [92]. It is mostly clinically silent, but it may also cause significant hematuria, proteinuria, and varying degrees of renal insufficiency [92]. Antiviral therapy along with interferon alfa is associated with improvements in renal function in patients with HCV-associated IgAN, which can support a pathogenic link between the two disorders [89,95,96].

  **6.**  **Fibrillary/Immunotactoid Glomerulopathy**

HCV patients have also been reported to have two less common glomerular diseases: fibrillary glomerulopathy and immunotactoid glomerulopathy [97,98,99,100]. Both conditions are characterized by the deposition of non-amyloid fibrils, which are believed to be composed of polymerized polyclonal immunoglobulin deposits [100,101,102]. The distinction between the two conditions is primarily based on the appearance, thickness, and arrangement of the deposited fibrils. In fibrillary, the fibrils are typically solid, 12–24 nm in diameter, and randomly arranged [97,103]. In contrast, immunotactoid fibrils are microtubular, greater than 30 nm in diameter, and exhibit a parallel arrangement [104].

Histopathological examination characteristically reveals extracellular deposits of microfibrils in the mesangium and glomerular capillary walls which stain negative for Congo red stain [37,105,106]. Immunofluorescence microscopy shows the presence of IgG, particularly IgG4, and C3 in the lesions [105]. Electron microscopy reveals the different-sized fibrils that help to differentiate the two conditions [103,107].

The typical presenting signs are those of nephritic syndrome, like hematuria, hypertension, and renal insufficiency, along with nephrotic range proteinuria [108,109,110].

##### Diagnosis of HCV-Related Glomerulopathies

HCV-infected patients often experience renal complications, which may include proteinuria, microscopic hematuria, hypertension, acute nephritis, and nephrotic syndrome with or without a reduction in glomerular filtration rate (GFR) [37,111]. Additionally, approximately 30% of these patients exhibit the triad of purpura, asthenia, and arthralgia [33]. Screening HCV-positive patients with renal complications should involve testing for anti-HCV antibodies and HCV RNA, along with evaluations for microalbuminuria, microscopic hematuria, RF, cryoglobulinemia, complement levels, and hypertension [37]. There are no universally accepted clinical diagnostic criteria for mixed cryoglobulinemia, but patients often present with “Meltzer’s triad” [64,112]. Indicators suggesting underlying cryoglobulinemic glomerulonephritis may include skin purpura, HCV infection, known autoimmune disorders, monoclonal gammopathy, or hematological malignancies [64]. Evidence of complement consumption, such as low serum C4 levels, further increases the likelihood of cryoglobulinemic glomerulonephritis [64,113].

Serologic testing for cryoglobulins requires that serum preparation be conducted at 37 °C to prevent premature immune complex precipitation [45]. The serum is stored at 4 °C for up to seven days, with daily inspections for any precipitate [45]. It is then centrifuged in a Wintrobe tube to measure the cryocrit, which indicates the percentage of cryoglobulins in the serum [45]. A cryocrit of 2% or higher is considered positive. Subsequently, the cryocrit is typed using immunofixation. In some chronic HCV patients with mixed cryoglobulinemia, HCV antibodies may be present while HCV RNA is undetectable in the plasma [45,114]. In such cases, testing the cryocrit for HCV RNA is recommended [45,114].

According to the KDIGO (Kidney Disease: Improving Global Outcomes) 2022 guidelines, patients with HCV infection who present with typical signs of immune-complex glomerulonephritis—such as hematuria, reduced C4 levels, circulating cryoglobulins, systemic manifestations of cryoglobulinemia, and the presence of RF, along with a stable GFR—can begin DAA therapy without a kidney biopsy [115]. However, if GFR or proteinuria worsens after treatment, or if immunosuppressive therapy is being considered, a kidney biopsy should be performed [115]. Additionally, patients who present atypically or have signs of rapidly progressive glomerulonephritis or severe nephrotic syndrome should also undergo a kidney biopsy [115].

#### 2.1.2. HCV-Related Vasculitis

HCV-related vasculitis affecting the kidneys includes cryoglobulinemic vasculitis and PAN. Cryoglobulinemic vasculitis and mixed cryoglobulinemia are the most common extrahepatic manifestations of chronic HCV infection [45]. HCV-associated mixed cryoglobulinemia typically involves Type I MPGN, which often indicates a poor clinical prognosis [33,64,116]. On the other hand, PAN is less frequently associated with HCV and presents as renal microaneurysms leading to clinical features such as hypertension in affected individuals [117,118].

##### Polyarteritis Nodosa

Secondary PAN is a medium-sized necrotizing vasculitis typically linked to HBV, but a few cases of PAN have been reported in HCV patients as well [119,120,121,122,123]. According to previous literature on patients with PAN, HCV antibodies were found in 20% of cases, while HCV RNA was detected in 5% [124]. In a study conducted by Saadoun et al. between 1990 and 2009, which aimed to analyze the main characteristics and outcomes of PAN-type vasculitis associated with HCV, it was reported that 31 (19.3%) out of the total 161 patients included in the study had PAN [125]. Similar findings were reported in a study conducted by Cacoub et al. on HCV-infected patients presenting with vasculitic syndromes [117].

As compared to the more common HCV-related vasculitis, that is, cryoglobulinemic vasculitis, PAN clinically presents as a more life-threatening vasculitis with severe multifocal sensorimotor mononeuropathies, as opposed to distal moderate sensory polyneuropathies, along with malignant hypertension, cerebral angiitis, ischemic abdominal pain, and microaneurysms in the kidneys and liver but lower rates of arthralgias, purpura, and chronic hepatitis activity [124,125,126,127]. In laboratory analysis, there is evidence of elevated acute phase reactants such as ESR and CRP, along with a higher incidence of reported renal insufficiency [126].

#### 2.1.3. Treatment of HCV-Related Glomerulopathies and Vasculitis

There are three potential strategies for treating HCV-associated glomerulopathies and vasculitic renal disease: etiologic/antiviral, pathogenetic, and symptomatic therapies [36].

The latest KDIGO 2022 guidelines [115] for HCV treatment in renal disease state the following: (a) Patients with HCV-associated glomerulonephritis who maintain stable kidney function and do not present with nephrotic syndrome should receive treatment with DAAs as the first-line option before exploring other therapies; (b) in patients undergoing a cryoglobulinemic flare or rapidly progressive glomerulonephritis, it is advised to use both DAAs and immunosuppressive agents, with the possible addition of plasma exchange. The choice to employ immunosuppressive agents in patients with nephrotic syndrome should be tailored to each individual (not graded); and (c) for patients with histologically active HCV-associated glomerulonephritis who do not respond to antiviral therapy, particularly those with cryoglobulinemic kidney disease, immunosuppressive therapy is advised. Rituximab is suggested as the first-choice immunosuppressive treatment [115].

  **1.**  **Etiologic/Antiviral therapy**

HCV does not have a long-term reservoir in the body, making it possible to completely and permanently cure the infection with antiviral treatment, unlike HBV and HIV [33]. Testing for HCV RNA 12 weeks or more after completing treatment is crucial. If HCV RNA is undetectable or unquantifiable at that time, it is considered a sustained virologic response (SVR), indicating a virologic cure. Virologic relapse is exceedingly rare 12 weeks or more after treatment. However, for patients with elevated ALT levels beyond the normal range, it may be beneficial to repeat quantitative HCV RNA testing at 24 weeks or later [128,129].

  (a) Interferons and Ribavirin: Combination of standard or pegylated interferon-α and ribavirin was historically the basis for HCV drug therapy [130]. These treatments needed to be administered for 6–12 months and had suboptimal efficacy of less than 50% [130]. Ribavirin is linked to several adverse events that can limit treatment, with hemolytic anemia being particularly notable [131]. The combination therapy of interferon and ribavirin frequently caused severe side effects, including neuropsychiatric changes, hematologic abnormalities, flu-like symptoms, and autoimmune toxicities [130].

Even in the era of all-oral DAAs, ribavirin continues to play a significant role in HCV treatment [132]. It is especially useful when DAAs have a low resistance barrier or when patients have characteristics that make achieving a cure more challenging such as in the setting of decompensated cirrhosis and prior DAA failures [132,133,134]. Ribavirin is generally well tolerated as part of all-oral DAA regimens, and the incidence and severity of anemia are significantly reduced and easier to manage without interferon [132].

  (b) Direct-acting antiviral agents (DAAs): The treatment of individuals with chronic HCV infection has been transformed by the introduction of HCV-specific antiviral therapies known as DAAs [33,130]. Improvements in GFR, reduction in proteinuria and hematuria, and a shortened treatment duration of 8–12 weeks with minimal side effects are key benefits of DAA therapy [94,135,136,137]. Combining two or more DAAs from different classes has increased SVR rates from approximately 50% to over 90% in the majority of patients with chronic HCV infection [33,138]. Selecting the appropriate DAA regimen is influenced by factors such as HCV genotype, prior treatment history, estimated glomerular filtration rate (eGFR), stage of hepatic fibrosis, and eligibility for kidney and liver transplantation [22,115]. According to the KDIGO 2022 guidelines, all individuals with chronic HCV, including those with glomerulonephritis, should receive treatment with DAAs in the same manner as those without glomerulonephritis [115]. Considering the resolution of hematuria, proteinuria, and the enhancement of GFR in patients with HCV-associated glomerulonephritis following HCV RNA clearance with DAAs, antiviral therapy using DAA regimens should be regarded as the primary treatment option for patients who do not have nephrotic syndrome and have relatively stable kidney function [115]. DAA therapy development is based on mapping the HCV genome, which includes non-structural (NS) proteins and the NS5B RNA polymerase involved in its replication cycle, leading to four classes of DAAs targeting specific viral components [115,139]. These classes include NS3/4A protease inhibitors (PIs) (have suffix “-previr”), NS5B nucleoside polymerase inhibitors (NPIs) (have suffix “-buvir”), NS5B non-nucleoside polymerase inhibitors (NNPIs) (have suffix “-buvir”), and NS5A inhibitors (have suffix “-asvir”) [115,139]. DAAs are often used in combination therapies to boost effectiveness and minimize the risk of viral resistance [115]. While some regimens are pangenotypic and effective against all HCV genotypes, others are genotype-specific, requiring genotype determination before starting DAA treatment [115].

When used in combination with ribavirin, sofosbuvir achieved excellent cure rates and resulted in notably fewer side effects [130,140,141]. The field swiftly progressed to combination DAA therapies, which enable regimens free of interferon and ribavirin and are highly well tolerated [130]. As per the latest Infectious Diseases Society of America and the American Association for the Study of Liver Diseases (AASLD-IDSA) guidelines, a simplified algorithm for treating HCV in treatment-naive adults without cirrhosis recommends pangenotypic direct-acting antiviral regimens, which include either 8 weeks of glecaprevir (300 mg)/pibrentasvir (120 mg) taken with food or 12 weeks of sofosbuvir (400 mg)/velpatasvir (100 mg) [129]. These regimens have been proven safe for use in CKD patients [115].

HCV-related glomerular disease can be challenging to manage with antiviral therapies, especially in cases involving proliferative or sclerotic damage [142]. Additionally, the effectiveness of DAAs in treating HCV-associated cryoglobulinemic vasculitis is limited, as these drugs cannot address the immune-mediated processes once they have been activated [142]. Nevertheless, clearing HCV RNA from the serum allows clinicians to use immunosuppressive treatments without worrying about HCV replication [142].

  **2.**  **Pathogenetic therapy/immunosuppressive therapy**

In patients with symptomatic mixed cryoglobulinemia, DAAs may not always be adequate [143]. Studies have shown that mixed cryoglobulinemic vasculitis, nephritis, and peripheral neuropathy may not significantly improve even after achieving SVR with DAA treatment [143,144]. When DAAs alone do not adequately control cryoglobulinemic symptoms, immunosuppressive therapy becomes necessary [115,145]. Given the connection between HCV infection and immune responses impacting the glomeruli, treatment strategies like immunosuppressive agents, high-dose corticosteroids, and plasma exchange have been employed for severe HCV-related glomerulopathies [36].

As already mentioned above, as per the KDIGO 2022 guidelines, it is advised to use both DAAs and immunosuppressive agents in patients undergoing a cryoglobulinemic flare or rapidly progressive glomerulonephritis [115]. Rituximab is currently the primary immunosuppressive medication used for treating HCV-related glomerulonephritis [115].

Rituximab is a chimeric monoclonal antibody combining human and mouse elements that binds to the CD20 antigen on the surface of B cells, selectively targeting these cells [146]. A key pathogenic aspect of mixed cryoglobulinemia, including cryoglobulinemic glomerulonephritis, is the chronic activation of B lymphocytes by HCV, which leads to extensive autoantibody production due to the HCV-induced reduction in the activation threshold of these cells [115]. Rituximab disrupts the production of cryoglobulins, monoclonal IgM, and the deposition of immune complexes in the kidneys [115]. In a prospective, single-center open study conducted by Roccatello D, long-term outcomes of administering rituximab to patients with severe mixed cryoglobulinemia were assessed [147]. Cryoglobulinemic nephropathy showed significant improvement during the follow-up, beginning as early as the second month after rituximab treatment (serum creatinine levels decreased from 2.1 ± 1.7 to 1.5 ± 1.6 mg/dL, *p* ≤ 0.05; 24 h proteinuria reduced from 2.3 ± 2.1 to 0.9 ± 1.9 g/24 h, *p* ≤ 0.05). No significant side effects were noted. Re-induction with rituximab was performed in 9 patients who relapsed after an average of 31.1 months (range 12–54 months), and beneficial effects were observed again. The 6-year survival rate was 75%, and there was a roughly 60% chance of staying symptom-free for 10 years without further therapy after just one “4 + 2” infusion cycle [147].

It is important to exercise caution with rituximab, as it has been linked to severe infectious complications [148]. Infectious episodes have been commonly reported in a vulnerable subgroup of patients, specifically those over 70 years of age, with a GFR of less than 60 mL/min per 1.73 m^2^, and those on concurrent high-dose corticosteroids, resulting in fatal outcomes for some individuals [149]. While reactivation of HCV is rare with rituximab therapy, reactivation of HBV is more commonly observed [148,150]. B-cell-depleting therapies, such as rituximab, carry a high risk of reactivation in both HBsAg-positive and HBsAg-negative but anti-HBc-positive patients; therefore, it is recommended that they undergo routine HBV screening using HBsAg and anti-HBc testing [150,151,152]. The American Gastroenterological Association (AGA) advises prophylaxis for patients undergoing high-risk and moderate-risk immunosuppressive therapies, with the recommendation to continue prophylaxis for 6 months after stopping the immunosuppressive treatment [153]. For B-cell-depleting therapies such as rituximab, it is advised that antiviral prophylaxis be maintained for 12 months following the final dose of the medication [153].

Corticosteroids form the foundation of treatment for PAN and are often paired with cyclophosphamide to manage severe and life-threatening complications [123]. Additionally, there are case reports highlighting the successful use of rituximab in treating a critical instance of HCV-associated PAN [122].

For many years, plasma exchange has been regarded as the preferred treatment for mixed cryoglobulinemia syndrome, whether or not the kidneys are affected [142]. The goal of this approach is to remove circulating cryoglobulins from the plasma and reduce the deposition of immune complexes in the kidneys [142]. To manage glomerular infiltration, steroid pulses or low-dose steroids have also been used [36,37,154]. Due to its ability to inhibit B lymphocytes and decrease cryoglobulin production, cyclophosphamide has been recommended for HCV-associated glomerulopathies [155]. It has been used successfully in this patient group, but the risk of HCV infection flare-ups and increased HCV RNA levels should always be considered [155]. Mycophenolate mofetil could warrant further investigation alongside conventional or selective immunosuppressive drugs [156]. Mycophenolate mofetil (MMF) is a more targeted treatment for inhibiting lymphocyte proliferation and function compared to cyclophosphamide, making it a safer option for inducing remission in cryoglobulinemic vasculitis [157].

  **3.**  **Symptomatic therapy:**

The primary clinical features of HCV-associated glomerulopathies include proteinuria and hypertension [37]. Therefore, renal protective treatment with antihypertensive and antiproteinuric agents, such as renin-angiotensin system inhibitors (including angiotensin-converting enzyme inhibitors and/or angiotensin II receptor blockers), should be administered as necessary [115,158,159]. Additional medications, such as systemic vasodilators, diuretics, and lipid-lowering agents, have demonstrated favorable outcomes in the symptomatic management of HCV-related renal disease [36].

### 2.2. Hepatitis C and Renal Stones

A recent cross-sectional study was conducted by Chen et al. using the National Health and Nutrition Examination Survey (NHANES) database to explore the effects of HCV on renal stone formation [160]. A total of 13,262 people were included in the study from the year 2007 to 2018. The study reported that participants with HCV infection were more likely to develop kidney stones. Thus, according to the study, HCV infection may be considered a risk factor for the formation of kidney stones in US women. The study could not prove a causal relationship between HCV infection and renal stone formation. Further studies are required to see the association between renal stones and HCV and the mechanisms responsible for the formation of renal stones in HCV patients [160]. Renal stone formation has also been reported in HCV-infected patients as a result of interferon and ribavirin therapy [161].

### 2.3. Chronic Kidney Disease in HCV-Infected Patients

Since its discovery, HCV has been linked to CKD [162]. The viral infection can either contribute to the development of CKD or result from it [163]. Individuals with pre-existing chronic kidney conditions face an increased susceptibility to acquiring HCV infection [36]. CKD is estimated to impact approximately 35.5 million people in the United States [164]. Up to 10% of HCV-infected patients in the United States are affected by CKD [31,130].

A 2015 meta-analysis by Park et al. revealed that people with HCV had a 23% higher risk of developing CKD compared to those without the infection (risk ratio = 1.23; 95% CI: 1.12–1.34) [165]. In a systematic review conducted by Fabrizi et al. in 2015, 23 studies involving 2,842,421 patients were deemed eligible, and separate meta-analyses were performed based on specific outcomes [166]. The combined results from longitudinal studies (*n* = 9 studies; 1,947,034 unique patients) indicated a significant association between positive HCV serologic status and an increased risk of developing CKD [166]. A 2016 meta-analysis by Min Li and colleagues, which included 12 longitudinal studies, also demonstrated a heightened risk of CKD associated with HCV, with an adjusted hazard ratio (aHR) of 1.45 and a 95% CI of 1.23–1.71 [167]. In another systematic review conducted by Fabrizi et al. in 2018 [168], examining 4,072,867 patients demonstrated a significant association between positive anti-HCV serologic status and an increased risk of CKD. The summary estimate for the adjusted hazard ratio with HCV across these studies was 1.54 (95% CI, 1.26; 1.87, *p* < 0.001). Meta-regression analysis revealed that aging (*p* < 0.0001) and longer follow-up duration (*p* < 0.0001) were associated with a higher risk of CKD in HCV-positive individuals. A relationship between anti-HCV positive serologic status and the frequency of proteinuria was also noted (aOR-1.63, 95% CI, 1.29–2.05, *p*< 0.001) based on 10 studies involving 315,404 unique patients [168]. These studies support the link between HCV infection and the onset of CKD, as well as the accelerated progression of CKD to end-stage renal disease (ESRD) requiring either transplantation or HD.

HIV and HCV co-infection is common, due to their similar routes of transmission, particularly among high-risk populations such people who inject drugs, MSM, and heterosexuals [169]. The patients with HIV and HCV co-infection tend to experience worse hepatic and extrahepatic outcomes, including an elevated risk of developing CKD [170,171,172]. Numerous studies have addressed the renal complications associated with HIV-HCV co-infection [172,173,174]. However, a detailed discussion of this topic is beyond the scope of our paper.

#### 2.3.1. Dialysis Patients and HCV Infection

The prevalence of HCV infection in patients undergoing HD is higher than in the general population and is linked to the duration of time spent on HD [115]. Patients with ESRD who undergo HD are at a higher risk of contracting HCV due to their need for permanent vascular access and their frequent contact with potentially contaminated medical equipment [36,175]. This not only adversely affects patient survival and subsequent transplant outcomes but also creates a reservoir that spreads the infection within the community [43,176,177].

The global prevalence of HCV infection among patients on HD varies significantly, influenced by factors such as differing HD practices, including contact precaution methods, the number of blood transfusions, and the duration of HD treatment [26]. Despite regional variations, the two primary factors that contribute to the higher prevalence of HCV infection in HD patients are the number of blood transfusions and the patient’s age [26,178,179].

The most recent survey from the Dialysis Outcomes and Practice Patterns Study (DOPPS), conducted between 2012 and 2015, reported that the overall prevalence of anti-HCV antibodies in patients on HD across 21 countries was 9.9% [180]. A systematic literature search was performed in MEDLINE and Scopus through March 2021 to evaluate the worldwide prevalence of HCV infection, as well as the related risk factors and clinical outcomes among HD patients [181]. The analysis included 407 studies with a total of 1,302,167 participants. The pooled prevalence of HCV infection was found to be 21%, with the highest rates observed in Africa (28%) and low-income countries (48.5%). A significant decrease in prevalence was noted over time, which was inversely correlated with a country’s GDP and total population. Factors linked to HCV positivity included younger age, longer duration on dialysis, increased number of blood transfusions, and reuse of dialyzers. The pooled unadjusted hazard ratio for all-cause mortality in HCV-infected patients, compared to those without HCV, was 1.12 (95% CI 1.03–1.22), while the adjusted hazard ratio was 1.21 (95% CI 1.12–1.30) [181].

In a recent systematic review and meta-analysis led by Momo-R et al. to estimate the global epidemiology of hepatitis C among dialysis patients, 634 studies involving 392,160 participants were included [182]. The overall case fatality rate for HCV was found to be 38.7% (95% CI = 28.9–49). The global prevalence of HCV infection in the dialysis population was 24.3% (95% CI = 22.6–25.9). When analyzed by UNSD (United Nations Statistics Division) for region, country, type of dialysis, and HCV diagnostic methods, Eastern Europe exhibited the highest prevalence at 48.6% (95% CI = 35.2–62), Indonesia showed a prevalence of 63.6% (95% CI = 42.9–82), HD patients had a prevalence of 25.5% (95% CI = 23.8–27.3), and anti-HCV was detected in 24.5% (95% CI = 22.8–26.2) of the cases. The high prevalence and case fatality rate of HCV infection among dialysis patients, especially those on HD, underscored the need for strict infection control measures in HD units [182].

HCV is primarily transmitted through parenteral routes, particularly via percutaneous exposure to blood [115]. Significant decreases in incidence were observed after the introduction of HCV screening for blood donors and the reduced need for blood transfusions following the use of erythropoiesis-stimulating agents [183]. As a result, nosocomial transmission has become the primary mode of HCV spread within dialysis units [183]. Multiple studies have corroborated nosocomial transmission in these settings through epidemiological and phylogenetic data obtained via viral sequencing [25,115]. Even in the present day, lapses in infection control practices and universal precautions have resulted in HCV outbreaks within dialysis centers [184,185]. Dialysis patients infected with HCV face high mortality rates, being six times more likely to die from liver disease and more likely to be hospitalized and tending to have lower quality-of-life scores [186]. In response, the CDC issued guidelines to enhance infection control practices to prevent HCV transmission in dialysis units [187].

The KDIGO 2022 guidelines for detection, evaluation, and prevention of HCV transmission in HD units are outlined as follows [115]:

1. Regular HCV screening in in-center HD patients should be conducted every six months using immunoassay or NAT [115].
2. It is suggested that serum alanine aminotransferase (ALT) levels be checked when patients begin in-center HD or transfer from another facility. Additionally, it is recommended that ALT levels in HD patients be monitored monthly [115].
3. HD facilities should strictly follow standard infection control procedures, including hygienic measures that effectively prevent the transfer of blood and blood-contaminated fluids between patients to avoid the transmission of blood-borne pathogens.
  3a. Regular observational audits of infection control practices in HD units are recommended.
  3b. It is recommended that dedicated dialysis machines should not be used exclusively for HCV-infected patients.
  3c. Isolation of HCV-infected HD patients is not suggested.
  3d. Reusing dialyzers for HCV-infected patients is suggested, provided that standard infection control procedures are strictly followed [115].
4. HD centers should monitor and track all HCV test results to identify any new cases of HCV infections among their patients; when a new HCV case is identified and likely related to dialysis, aggressive measures should be implemented to improve hand hygiene (including proper glove use), injection safety, and the cleaning and disinfection of the environment [115].
5. Strategies to prevent HCV transmission within HD units should focus on adherence to standard infection control practices rather than primarily relying on the treatment of HCV-infected patients (Not Graded) [115].

#### 2.3.2. Kidney Transplantation and HCV Infection

Kidney transplantation remains the most effective treatment for advanced kidney disease, offering significant improvements in both quality of life and survival, even for patients on dialysis who are infected with the HCV [115,188,189]. The prevalence of HCV infection in pre-kidney transplantation patients has been reported to be as high as 40% [36,138]. However, more recent studies show a decrease in prevalence due to the adoption of preventive measures in CKD patients, although it still fluctuates between 3% and 80% in different countries [43,138]. HCV infection remains prevalent among patients with CKD, with estimates indicating that about 10% of individuals awaiting kidney transplants are chronically infected with the virus [188,190].

HCV infection in kidney transplant recipients is linked to higher rates of morbidity and mortality and contributes to various hepatic and extrahepatic complications [191]. After kidney transplantation, chronic hepatitis and HCC are the predominant liver diseases, while fibrosing cholestatic hepatitis, though severe, is less common [188,191,192]. HCV-related liver disease heightens the risk of acute rejection, de novo glomerulonephritis, thrombotic microangiopathy, proteinuria, and new-onset diabetes [130,193,194,195]. In HCV-positive patients following kidney transplantation, various glomerular lesions have been identified. The most frequent are de novo or recurrent MPGN, often but not always associated with cryoglobulinemic disease, and MN [190,196]. Chronic allograft nephropathy is another adverse outcome that occurs more frequently in recipients who are anti-HCV-positive compared to those who are matched and HCV-negative [190,197,198].

Before the advent of DAA therapy, the survival rates of kidney transplant patients with persistent HCV viremia were lower than those of HCV-negative transplant recipients but were still better than if they had continued on dialysis [115,190,199,200]. The approval of DAAs for HCV treatment in both dialysis and kidney transplant patients enables nearly all patients to achieve successful HCV clearance either before or after transplantation [115]. Those who attain SVR before transplantation do not experience relapse after the procedure, even with the use of strong immunosuppressive medications [115,201,202]. Therefore, eligible patients should be considered for kidney transplantation irrespective of their HCV status [115].

The need for transplant organs significantly surpasses their availability [203]. According to the Organ Procurement and Transplantation Network (OPTN), over 90,000 individuals are currently on the waiting list for a kidney transplant [203]. To address the organ shortage issue, one approach has been the utilization of organs from donors infected with HCV [204]. The development of new, highly effective interferon-free treatments with success rates exceeding 95% has enabled the use of HCV-positive organs, particularly in light of the significant rise in mortality associated with drug overdoses, especially opioids, in the United States [204,205]. Historically, organs from HCV-positive donors were discarded at nearly three times the rate of those from HCV-negative donors [206]. Given the high effectiveness of DAAs, utilizing kidneys from deceased donors infected with HCV could potentially increase kidney transplantation rates [207]. Gorden et al. conducted a systematic review for the 2022 KDIGO Clinical Practice Guideline on HCV, assessing the safety and efficacy of kidney transplants from HCV-positive donors to uninfected recipients (D+/R−), followed by DAA therapy. The review, which included 16 studies involving 557 patients, found high rates of SVR, low incidence of adverse events, and excellent patient and allograft survival one-year post-transplant [207]. The findings suggested that kidney transplants from HCV-positive donors to uninfected recipients, followed by DAA treatment, are both safe and effective [207].

As per the KDIGO 2022 guidelines, all kidney transplant candidates with HCV should undergo an evaluation to assess the severity of liver disease and the presence of portal hypertension prior to being accepted for kidney transplantation [115]. It is recommended that patients with HCV, compensated cirrhosis, and no portal hypertension proceed with isolated kidney transplantation, while those with decompensated cirrhosis or significant portal hypertension (e.g., hepatic venous pressure gradient ≥ 10 mm Hg or evidence of portal hypertension on imaging or examination) should be considered for simultaneous liver-kidney transplantation [115]. The guidelines recommend that all kidney transplant candidates with HCV be considered for DAA therapy, whether before or after transplantation [115]. For HCV-infected kidney transplant candidates with a living kidney donor, it is suggested that treatment be considered before or shortly after transplantation, depending on the expected timing of the transplant [115].

Clinical trials specifically conducted in transplant recipients have demonstrated that DAAs can be safely administered after kidney transplantation, achieving excellent cure rates without leading to allograft dysfunction or acute rejection [208,209]. The incidence of HCV-related post-transplant glomerulonephritis is also expected to be lower in transplant recipients treated with DAAs [130].

#### 2.3.3. Treatment of HCV in CKD Patients

According to the KDIGO 2022 guidelines, DAAs have been accepted as a well-tolerated treatment for HCV in patients across all CKD stages, including those on dialysis and kidney transplant recipients, without requiring dose adjustments [115]. Pangenotypic DAA regimens, including sofosbuvir-based options as well as genotype-specific regimens, are safe and effective for patients with advanced CKD and kidney transplant recipients [115]. Treatment selection can be based on local practices and the availability of specific DAAs [115]. If pangenotypic regimens are not accessible, it is important to determine the patient’s genotype before initiating DAA therapy [115].

In a phase 3, prospective randomized trial known as the C-SURFER study, Roth et al. evaluated elbasvir (HCV-NS5A inhibitor) and grazoprevir (HCV-NS3/NS4A protease inhibitor) in both treatment-naive and treatment-experienced patients with HCV genotype 1 infection and stage 4–5 CKD [210]. The results showed an impressive SVR rate of 99% with a low incidence of adverse effects. The once daily regimen of grazoprevir and elbasvir for 12 weeks was highly effective and well tolerated in these patients [210].

In a cohort of 403 patients treated with DAA regimens, changes in eGFR were analyzed by Copolla et al. [211]. The overall sustained virological response rate was 98%, and the proportion of patients with an eGFR greater than 60 mL/min/1.73 m^2^ increased significantly from 83.1% at baseline to 87.8% at 12 weeks post treatment. Additionally, 148 patients experienced an improvement in eGFR, defined as an increase of at least 10 mL/min/1.73 m^2^ from baseline [211].

### 2.4. Nephrotoxicity of HCV Drugs

HCV virus treatment-related nephrotoxicity is caused by the drugs used to treat the condition. DAAs are generally regarded as non-nephrotoxic, with extensive studies demonstrating very low incidences of acute kidney injury (AKI) associated with their use [212]. Nevertheless, limited case series have associated DAA use to be associated with development of lupus-like glomerulonephritis and podocytopathies, and there have been case reports of acute interstitial nephritis in this patient population [213,214,215,216].

Ribavirin’s renal toxicity has not been reported and is unlikely when used as a monotherapy [217].

In a recent study involving 3264 patients, researchers monitored changes in kidney function measured by eGFR during HCV treatment with DAAs [218]. They observed that kidney function declined in patients with the mildest kidney disease at the start of the treatment but improved in those with more severe kidney disease. These changes persisted even after achieving an SVR indicating the virus was no longer detectable [218].

Renal clearance is the major elimination pathway for sofosbuvir [219]. The HCV TARGET study, led by Saxena et al., investigated the relationship between initial renal impairment and the efficacy and safety of sofosbuvir-based regimens in a multinational cohort of 1893 patients with chronic hepatitis C. These patients had varying levels of renal function, as indicated by their eGFR [220]. Most of the participants were infected with HCV genotype 1, and the treatments included combinations such as sofosbuvir/pegylated interferon/ribavirin, sofosbuvir/ribavirin, and sofosbuvir/simeprevir, with or without ribavirin. The study found that the overall SVR12 was consistent across different eGFR groups, with rates around 82–83%. However, patients with a lower baseline eGFR (≤45 mL/min/1.73 m^2^) experienced a higher incidence of renal function decline and serious adverse events, with rates at least 3.5 times higher than those with a baseline eGFR above 45 mL/min/1.73 m^2^ [220]. Similarly, another study focused on 1536 Asian patients with chronic HCV found that eGFR significantly declined by the end of the treatment but slightly improved 12 weeks post treatment [221]. This pattern was consistent across various DAA regimens except for sofosbuvir based treatments, where kidney function remained stable, particularly in liver transplant recipients [221].

Sofosbuvir was initially not approved for patients with severe renal insufficiency eGFR rate below 30 mL/min) or ESRD, but more recent studies have suggested sofosbuvir-based therapies to be safe in chronic hepatitis C patients with baseline normal or impaired renal function [222,223].

A recent study found that DAAs are highly effective and well tolerated in patients with ESRD and kidney transplant recipients, achieving SVR of over 96% HCV infection [224]. Occult HCV infection was rare, and no adverse clinical implications were associated with HCV RNA in peripheral blood mononuclear cells. While kidney function measured by serum creatinine and eGFR remained stable during antiviral therapy, it declined over four years. No instances of AKI were observed suggesting the progressive nature of CKD warrants further investigation to understand long-term kidney function post antiviral therapy [224].

## 3. Conclusions

Chronic HCV infection can lead to a spectrum of renal complications, ranging from mild proteinuria to severe glomerulonephritis, often necessitating dialysis or even kidney transplantation in advanced cases. The DAAs has significantly improved treatment outcomes, but challenges remain, particularly in managing immune-mediated renal diseases like mixed cryoglobulinemia and PAN. Immunosuppressive therapies, such as rituximab, are crucial in these scenarios. Optimizing treatment protocols and exploring new therapies for resistant cases, especially in patients requiring dialysis or post-transplant care, and in patients with concurrent severe renal impairment or those who are at high risk for adverse events will be vital in improving long-term patient outcomes.

## Data Availability

Data sharing is not applicable to this article, as no new data were created or analyzed in this study.
